# IMPROVING ACCESS OR INCREASING DISPARITIES? IMPLEMENTATION OF TELEHEALTH LITERACY SCREENING PROGRAM

**DOI:** 10.1093/geroni/igae098.1405

**Published:** 2024-12-31

**Authors:** Madison Boudreaux, Aleksandra Lindgren, Rhonda Bardales, Scott Wilks, Kathy Carstarphsen

**Affiliations:** UQ-Ochsner Clinical School, New Orleans, Australia; UQ-Ochsner Clinical School, New Orleans, Louisiana, United States; Louisiana State University, Baton Rouge, Louisiana, United States; Louisiana State University, Baton Rouge, Louisiana, United States; Tulane University, New Orleans, Louisiana, United States

## Abstract

The use of telehealth services is rising in the United States, especially since the pandemic, and continues to be explored as a method in removing barriers to accessing healthcare services. However, those living with vision, hearing, and cognitive impairments may be marginalized in the use of telehealth without an efficient and accurate way of measuring their use and comfortability of engaging with telehealth modalities. The Telehealth Literacy Screening Tool (TLST) was created to measure telehealth literacy, access, use of, and comfortability with technology. This study examined the differences between impairment groups to inform practitioners of potential barriers existing for those living with vision/hearing or cognitive impairments when using telehealth services. The TLST was administered to patients in an outpatient primary care clinic (N=128) over a 5-month period. Text message and email usage varied by group, with those living without visual/hearing or cognitive impairment reporting higher rates of use. Those with both visual/hearing and cognitive impairments had lower comfortability with opening apps than the group with either impairment, and lower comfortability with scrolling than the group with no impairment. The use of the TLST can help identify those patients with limits in telehealth literacy so their specific needs for education or other modalities can be met. This study strengthened the usefulness of the TLST in primary care practice and identified the disparity in telehealth usage among patients with hearing/visual or cognitive impairments.

